# *In vitro* feeding of nymphal *Haemaphysalis longicornis* supports moulting to adults and maintains *Coxiella*-dominated microbiome across life stages

**DOI:** 10.1016/j.crpvbd.2026.100426

**Published:** 2026-08-18

**Authors:** Abdul Ghafar, Bahar E. Mustafa, Mehran Khan, Charles Gauci, Ian Beveridge, Robin B. Gasser, Ard M. Nijhof, Abdul Jabbar

**Affiliations:** a Department of Veterinary Biosciences, Melbourne Veterinary School, The University of Melbourne, Victoria, Australia; b Institute for Parasitology and Tropical Veterinary Medicine, Freie Universität Berlin, Robert-Von-Ostertag-Str. 7, Berlin, Germany; c Veterinary Centre for Resistance Research, Freie Universität Berlin, Robert-Von-Ostertag-Str. 8, Berlin, Germany

**Keywords:** Artificial tick feeding system, Asian longhorned tick, *Coxiella*-like endosymbiont, *Haemaphysalis longicornis*, *In vitro* feeding, Tick microbiome, Trans-stadial persistence

## Abstract

Artificial tick feeding systems (ATFS) enable controlled investigation of tick biology under *in vitro* membrane-feeding conditions. However, their application to immature stages and microbiome structure during prolonged host-free maintenance remains poorly understood. Here, we extend a recently established ATFS to the nymphal stage of *Haemaphysalis longicornis*, an invasive tick of global significance and the primary vector of *Theileria orientalis* in Australia. We provide the first integrated assessment of feeding performance, post-feeding development and trans-stadial microbiome composition under *in vitro* membrane-feeding conditions. Nymphs were fed *in vitro* across five independent feeding units, achieving high attachment (80–100%) and engorgement success (83–100%), with moulting success of 90.5%. Developmental timing was consistent, with attachment occurring within 1–5 days, detachment within 4–10 days, and moulting 14–25 days post-detachment. Variation in engorgement weight reflected individual heterogeneity while demonstrating reproducible feeding performance. Microbiome profiling revealed a low-diversity bacterial community strongly dominated by a *Coxiella*-like endosymbiont (CLE) across all life stages. Although alpha diversity differed significantly between unfed nymphs and adults, overall community composition remained broadly stable from unfed nymphs through *in vitro*-fed adults to their larval progeny. This persisted during extended maintenance without exposure to a live vertebrate host, indicating maintenance of host-symbiont interactions. Collectively, these findings demonstrate that ATFS supports nymphal feeding, development to adults and trans-stadial dominance of CLEs in *H. longicornis* across stages. This work represents a key step toward establishment of a complete *in vitro* life-cycle system for future studies of tick physiology, symbionts, microbiome-pathogen interactions and control strategies, while advancing the principles of the 3Rs in tick research.

## Highlights

• Nymphal *Haemaphysalis longicornis* were successfully fed *in vitro*.
• High attachment, engorgement and moulting success were achieved.
• Artificial feeding supported successful moulting from nymphs to adults.
• *Coxiella*-like endosymbionts remained dominant across tick life stages.
• Findings support development of a complete artificial tick life-cycle system.

## Graphical abstract

**Figure:**
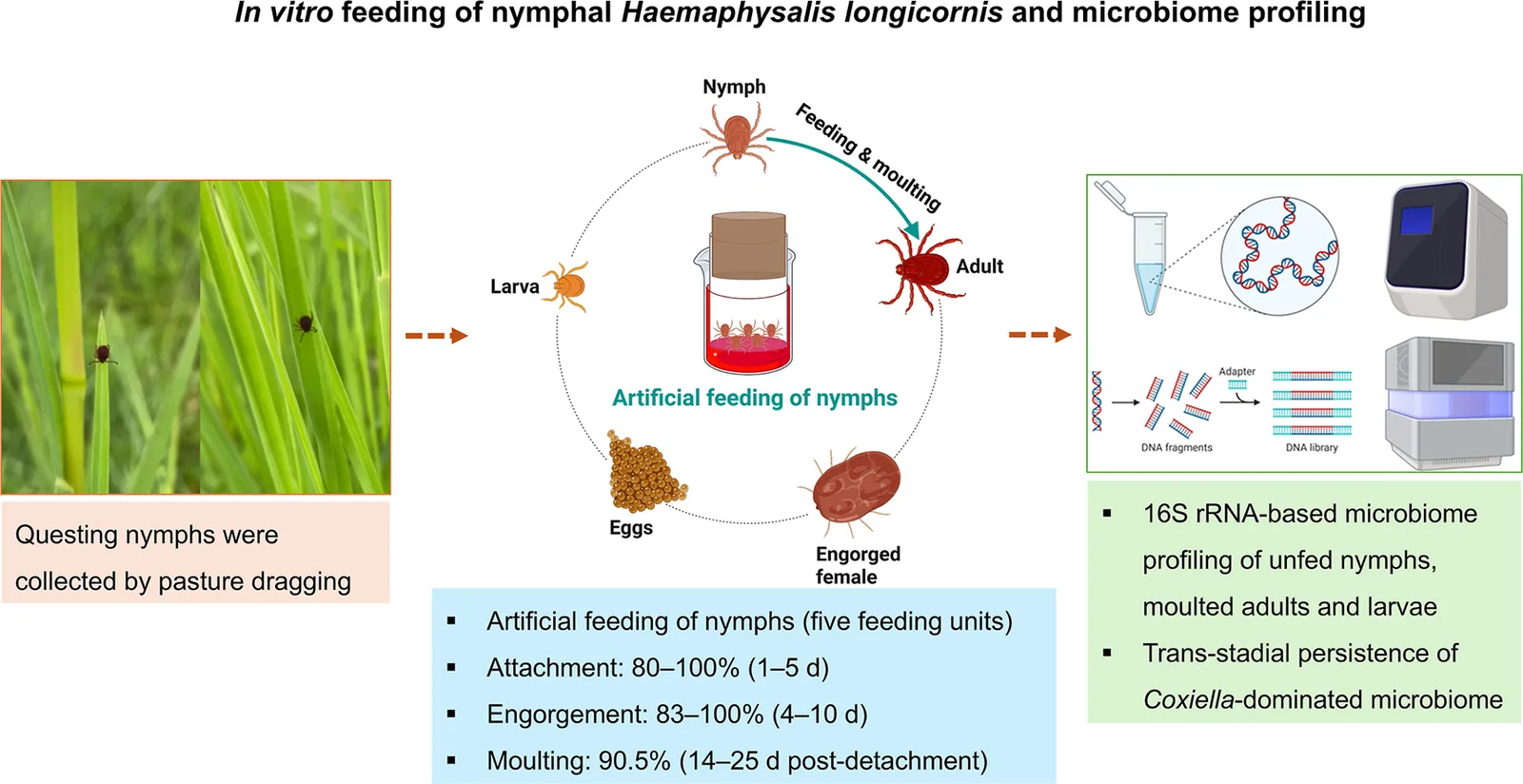

## 1 Introduction

Ticks are obligate haematophagous ectoparasites and are among the most important vectors of pathogens affecting both animals and humans (Jongejan and Uilenberg, 2004). They transmit a wide range of viruses, bacteria and protozoa, resulting in significant impacts on animal health, productivity and biosecurity (Jongejan and Uilenberg, 2004; de la Fuente et al., 2008). In addition to their role as vectors, ticks harbour complex microbial communities, including symbionts and potential pathogens, which are increasingly recognised as key contributors to tick physiology, development and vector competence (Bonnet et al., 2017).

Understanding tick feeding biology is central to elucidating pathogen transmission dynamics and identifying novel intervention strategies (Wu-Chuang et al., 2021). However, experimental studies have traditionally relied on animal-based feeding systems, which are labour-intensive, ethically constrained and difficult to standardise (Mustafa et al., 2026). As tick feeding is a prolonged and highly coordinated process requiring sustained host interaction (Sonenshine and Roe, 2014), reliance on live hosts introduces variability that limits reproducibility.

Artificial tick feeding systems (ATFS) provide a controlled alternative to feeding on live vertebrate hosts for investigating tick feeding, development and pathogen transmission, while supporting the principles of Replacement, Reduction and Refinement (3Rs) (Kröber and Guerin, 2007; Nijhof and Tyson, 2018; Mustafa et al., 2026). These systems can reproduce key features of natural feeding, including attachment dynamics, cement cone formation and characteristic feeding phases. However, robust and reproducible ATFS remain limited for immature tick stages, where attachment, feeding efficiency and subsequent development are often inconsistent or poorly characterised (Mustafa et al., 2026).

*Haemaphysalis longicornis* is an invasive and rapidly expanding tick species of major global concern, acting as a key vector of *Theileria orientalis* in Australia and contributing to emerging tick-borne disease risks in newly established regions (Heath, 2016). Originally distributed across East Asia, *H. longicornis* has expanded into Australasia, North America and Europe (Heath, 2016; Beard et al., 2018). Its ability to reproduce parthenogenetically in some populations, combined with a broad host range and ecological adaptability, has facilitated rapid population expansion and establishment in new environments. These traits, together with its capacity to reach high population densities, contribute to its growing importance in both endemic and newly invaded regions (Zhao et al., 2020).

The species is of particular veterinary significance due to its role as a vector of economically important pathogens, including *T*. *orientalis*. Once considered of limited pathogenicity, the emergence of virulent genotypes has resulted in substantial production losses across the Asia-Pacific region and beyond (Gebrekidan et al., 2020; Ghafar et al., 2026b). In addition, *H. longicornis* has been implicated in the transmission of a range of bacterial and viral agents, including pathogens of zoonotic importance such as severe fever with thrombocytopenia syndrome virus (Dabie bandavirus), highlighting its broader biosecurity relevance (Zhao et al., 2020). This highlights the importance of understanding its biology under controlled conditions to support improved control strategies.

In our recent work, we demonstrated that adult *H. longicornis* can be successfully fed under *in vitro* membrane-feeding conditions, achieving attachment, engorgement and complete reproductive output (Ghafar et al., 2026a). This established a promising platform for controlled investigations of tick feeding biology without reliance on live vertebrate hosts. However, the applicability of such systems to immature stages remains unresolved, representing a critical barrier to establishing a complete *in vitro* life-cycle model.

Nymphal feeding represents a critical transitional stage in the three-host life cycle of *H. longicornis*, linking larval development to reproductive adult stages. Successful *in vitro* feeding of nymphs, followed by moulting into adults, would therefore represent a key step toward the establishment of a complete *in vitro* life-cycle system. Achieving this would enable fully controlled, stage-spanning investigations of tick development, physiology and vector competence within a standardised experimental framework.

The tick microbiome plays an important role in nutrition, development and pathogen interactions (Narasimhan and Fikrig, 2015; Bonnet et al., 2017). Although blood-feeding and moulting influence microbial composition, it remains unclear whether artificial feeding systems maintain dominant symbiont-associated microbiome structure across life stages, particularly during prolonged maintenance following artificial feeding conditions where physiological stress and nutrient limitation may affect symbiont persistence. In particular, the stability of dominant bacteria such as *Coxiella*-like endosymbionts (CLEs) remains poorly understood. Evaluating whether core microbial associations are retained is important for assessing the biological relevance of ATFS-derived ticks.

The present study aimed to evaluate the performance of an ATFS applied to nymphal *H. longicornis*, assessing attachment, engorgement, engorgement weight and moulting success across independent feeding units to quantify feeding performance. We also characterised microbiome profiles of pre-fed starved nymphs, post-fed adults and larvae derived from *in vitro*-fed adults to assess microbial continuity across life stages. Collectively, this study addresses a key limitation in experimentally investigating tick biology by extending artificial feeding to immature stages of *H. longicornis* and evaluating both developmental performance and microbiome composition under artificial membrane-feeding conditions.

## 2 Materials and methods

### 2.1 Tick source and experimental design

*Haemaphysalis longicornis* nymphs were obtained from field-derived populations in Little Forest, New South Wales, Australia, and maintained under controlled laboratory conditions (20 ± 1 °C; ≥ 80% relative humidity) in desiccators until use, consistent with conditions described previously (Ghafar et al., 2026a). All nymphs originated from a single field population collected during a single sampling event in February 2025 and were subsequently maintained under identical laboratory conditions until experimentation. Five independent feeding experiments (feeding units; FUs 1–5) were conducted sequentially between April and June 2025. Individual FUs represented independent experimental runs, and comparisons among these units were interpreted descriptively. Between 12 and 26 nymphs were introduced per feeding unit, yielding a total of 86 nymphs across all experiments.

### 2.2 Artificial feeding system

The artificial feeding system was adapted from a previously established ATFS for adult *H. longicornis* (Ghafar et al., 2026a). FUs consisted of silicone membranes (∼30–40 μm thickness) supported by goldbeater’s skin and mounted on glass feeders as previously described. FUs were placed in glass beakers containing defibrinated bovine blood (Serum Australis, Australia) supplemented with glucose and adenosine triphosphate (ATP). To facilitate attachment, membranes were overlaid with raw cattle hair obtained during routine grooming of the University of Melbourne teaching herd at the Werribee campus. Feeding was conducted under controlled conditions, with blood maintained at 36 °C, ambient temperature at ∼27 °C, ≥ 80% relative humidity and 3.5% CO_2_, following our previously established artificial feeding conditions for adult *H. longicornis* (Ghafar et al., 2026a). Blood was replaced twice daily at 10–14 h intervals under aseptic conditions in a biosafety cabinet (SafeMate ECO+0.9 ABC, Italy). Before use, fresh blood aliquots were warmed to 37 °C in a water bath. During each blood replacement, the membranes were rinsed with sterile normal saline, dried, and the FUs were transferred to clean glass beakers. Between feeding cycles, the CO_2_ incubator (In-VitroCell Direct Heat CO_2_ Incubator, NuAire, USA) was cleaned with 70% ethanol, allowed to dry completely, and subjected to the built-in decontamination cycle before reuse.

### 2.3 Monitoring of feeding and developmental performance

FUs were inspected twice daily to record attachment, detachment and mortality. At each observation, ticks were counted collectively within each feeding unit according to their feeding status. Detached engorged nymphs were subsequently removed and recorded individually for engorgement-weight measurement, where possible, and monitoring of moulting. Attachment was defined as successful adherence to the membrane and initiation of feeding. Engorgement was identified by the visibly enlarged body of a fully fed nymph, whereas detachment was defined as its spontaneous release from the membrane. Detached engorged nymphs were collected immediately after detachment and transferred to desiccators maintained at 20 ± 1 °C and ≥ 80% relative humidity for monitoring of moulting. Feeding performance metrics were calculated as follows: attachment rate (%) = (number attached ÷ number introduced) × 100; engorgement rate (%) = (number engorged ÷ number introduced) × 100; engorgement success (%) = (number engorged ÷ number attached) × 100; and moulting success (%) = (number moulted ÷ number engorged) × 100. Moulting duration was calculated relative to the day of detachment of engorged nymphs. Engorgement weights were recorded for individual nymphs where possible using a precision balance.

### 2.4 Microbiome sequencing

Microbiome profiling was conducted across three life stages representing different points in the *in vitro* experimental pipeline: pre-fed nymphs; adults moulted from *in vitro*-fed nymphs; and larvae derived from adults. Pre-fed nymph samples (AG1-AG4) consisted of pools of five individuals. Adult samples (AG5-AG9) comprised one adult tick per sample, each derived from nymphs fed in feeding units 1–5, respectively. Larval samples (AG10-AG12) consisted of pools of approximately 50–60 individuals derived from eggs produced by adults that had moulted from nymphs fed in feeding units 2 and 3 and were subsequently fed under *in vitro* conditions. At the time of DNA extraction, unfed nymphs had been maintained without feeding for 12 months, while adults and larvae represented post-feeding stages maintained for approximately 7–8 months and 5–6 months, respectively. These differences were not experimentally imposed starvation treatments but reflected the sequential timing of feeding, moulting, reproduction and sample collection across life stages.

Prior to DNA extraction, ticks were surface-sterilised using 5% sodium hypochlorite (LabCo, Australia) for 5 min. Genomic DNA was extracted using Qiagen Blood and Tissue Kit (Qiagen, Hilden, Germany) according to the manufacturer’s guidelines, with the incubation step in proteinase K extended for up to 48 h. The V4 “hypervariable” region of the bacterial 16S rRNA gene was amplified using a universal primer pair (designated 515F-806R), and libraries were prepared using a two-step PCR protocol as described previously (Apprill et al., 2015; Parada et al., 2016; Ghafar et al., 2021). Libraries were sequenced on an Illumina NextSeq 1000 platform using NextSeq 1000/2000 P1 (600 cycles) kit. Negative controls (nuclease-free water) and positive controls (ZymoBIOMICS Microbial Community DNA Standard) were also included.

### 2.5 Statistical and microbiome data analysis

Differences among feeding units in attachment timing, detachment timing and moulting duration were assessed using the Kruskal-Wallis test. Where significant, pairwise comparisons were performed using Dunn’s tests with Bonferroni adjustment. Raw sequence data were processed using the QIIME 2 pipeline (version 2025.4). Demultiplexed paired-end reads were imported using a manifest-based approach and visualised to assess sequence quality. Primer sequences were removed using Cutadapt with degenerate primer matching. Additional trimming was performed to remove poly-G and poly-C artefacts, and alternative trimming strategies were evaluated to optimise sequence quality and retention.

Denoising, chimera removal and amplicon sequence variant (ASV) inference were performed using DADA2 with truncation lengths of 210 bp (forward reads) and 120 bp (reverse reads). Taxonomic classification was performed using a naïve Bayes classifier trained on the SILVA 138.2 reference database trimmed to the V4 region (515F-806R). ASVs assigned to chloroplasts, mitochondrial sequences and non-bacterial taxa (including host-derived sequences) were removed prior to analysis.

Data were analysed in R (RStudio, 2025.05.1 Build 513). Feeding and developmental performance were recorded and visualised using *ggplot2* and *ggpubr*. Given small sample sizes and non-normal distributions, non-parametric tests were applied throughout. Differences in engorgement weight among FUs were assessed using the Kruskal-Wallis test. Associations between continuous variables were evaluated using Spearman’s rank correlation.

Microbiome data were analysed using the phyloseq framework. Sequences from contaminants were identified and removed using the *decontam* package, applying a prevalence-based approach with a threshold of 0.3, selected to balance removal of control-enriched taxa while retaining biologically relevant sequences. Data were transformed to relative abundance and taxonomic composition was summarised at the genus level, with low-abundance taxa grouped as “Other” where appropriate.

Samples were rarefied to 181,000 reads per sample prior to alpha- and beta-diversity analyses to standardise sequencing depth across samples. Alpha diversity was assessed using the Shannon index, with differences among life stages evaluated using the Kruskal-Wallis test followed by pairwise Wilcoxon tests with Benjamini-Hochberg correction. Beta diversity was assessed using Bray-Curtis dissimilarity and visualised *via* principal coordinates analysis (PCoA), with differences evaluated using PERMANOVA. Homogeneity of multivariate dispersion was assessed using PERMDISP *via* the “betadisper” function (*vegan* package), with significance tested using permutation analysis (999 permutations).

## 3 Results

### 3.1 Feeding performance across FUs

A total of 86 nymphs of *H*. *longicornis* were introduced across five independent feeding units (FU1-FU5), with 12–26 individuals per unit. Overall, high levels of attachment were observed, ranging from 80.0% to 100%, with three of five feeding units achieving ≥ 88% attachment (Table 1). Representative stages of *in vitro* feeding and subsequent development are shown in Supplementary Fig. S1. Engorgement success, calculated relative to attached nymphs, was similarly high, with complete engorgement (100%) observed in FU1, FU2, FU4 and FU5, and a lower engorgement rate of 83.3% in FU3. When expressed relative to total introduced nymphs, engorgement rates ranged from 66.7% to 100%. Moulting success varied among feeding units, ranging from 75.0% (FU4) to 100% (FU2 and FU3). Overall, 67 of 74 engorged nymphs successfully moulted to adults, corresponding to a cumulative moulting success of 90.5%. These results demonstrate that the artificial feeding system consistently supported attachment, engorgement and subsequent development, although variability among feeding units was observed.

**Table 1:** Feeding performance and developmental outcomes of *Haemaphysalis longicornis* nymphs across five independent feeding units.

Feeding unit	Nymphs added	Attached *n* (%)	Engorged *n* (%)^a^	Moulted *n* (%)^b^	Attachment (days)	Detachment (days)	Moulting (days)	Mean engorgement weight ± SD (mg)
FU1	26	23 (88.5)	23 (100)	20 (87.0)	2–3	4–7	22–25	4.48 ± 0.65
FU2	18	18 (100)	18 (100)	18 (100)	2–3	5–8	14–19	4.28 ± 0.48
FU3	15	12 (80.0)	10 (83.3)	10 (100)	1–2	4–6	14–22	3.97 ± 0.93
FU4	15	12 (80.0)	12 (100)	9 (75.0)	1–5	5–9	20–24	3.90 ± 1.10
FU5	12	11 (91.7)	11 (100)	10 (90.9)	1–5	7–10	17–23	4.23 ± 0.78

*Abbreviations: n*, number of nymphs; SD, standard deviation.

a Percentages for engorged ticks are calculated relative to the number of ticks that successfully attached.

b Percentages for moulting are calculated relative to the number of engorged nymphs. Moulting period is expressed as days post-detachment.

### 3.2 Feeding dynamics and developmental performance

**Fig. 1:**
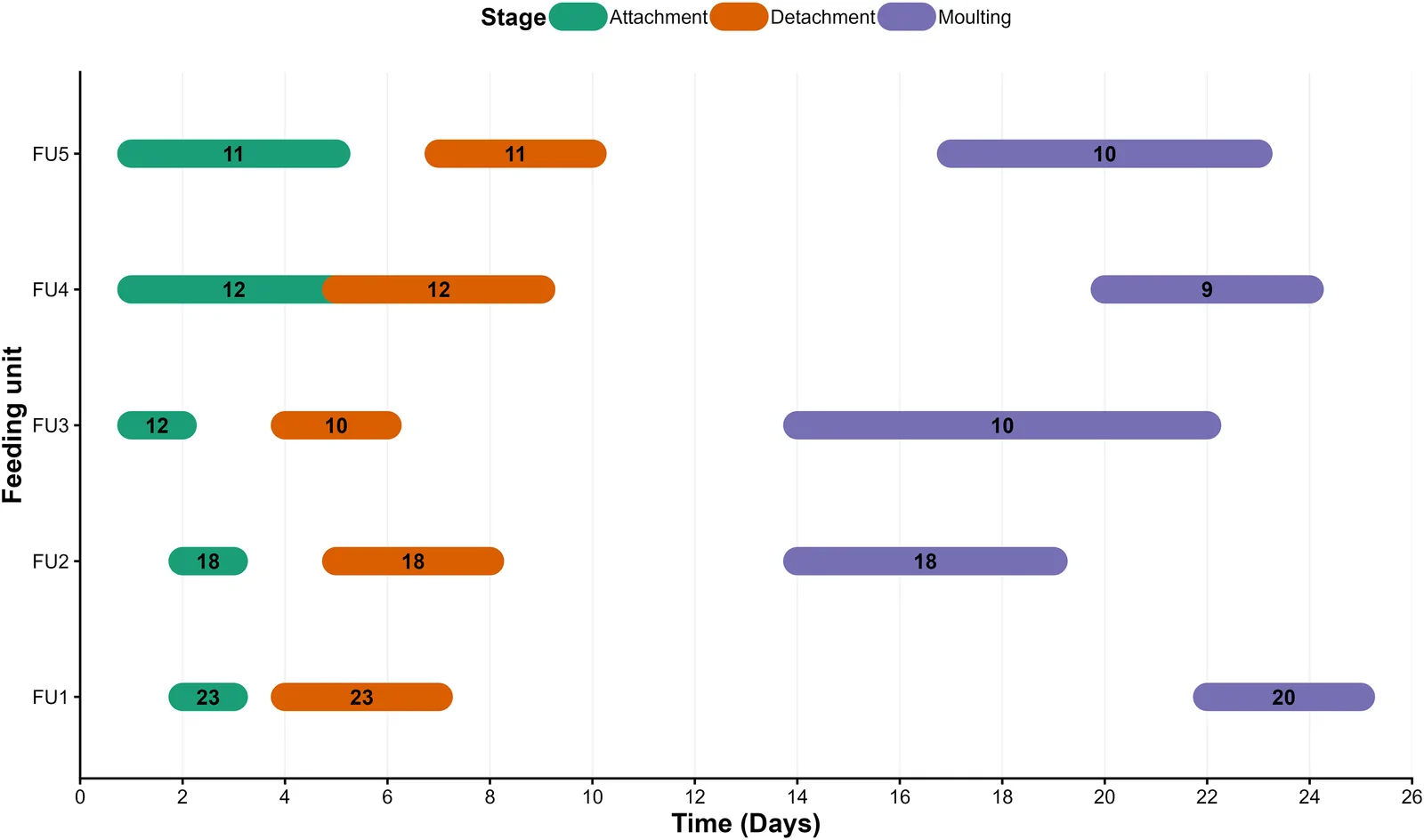
Life-stage progression and feeding outcomes of *Haemaphysalis longicornis* nymphs across five independent feeding units (FU1-FU5). Horizontal bars show the time range for attachment, detachment of engorged nymphs, and moulting. Values indicate the number of ticks completing each stage, relative to introduced, attached and engorged ticks. Moulting timing is shown relative to detachment of engorged nymphs.

**Fig. 2:**
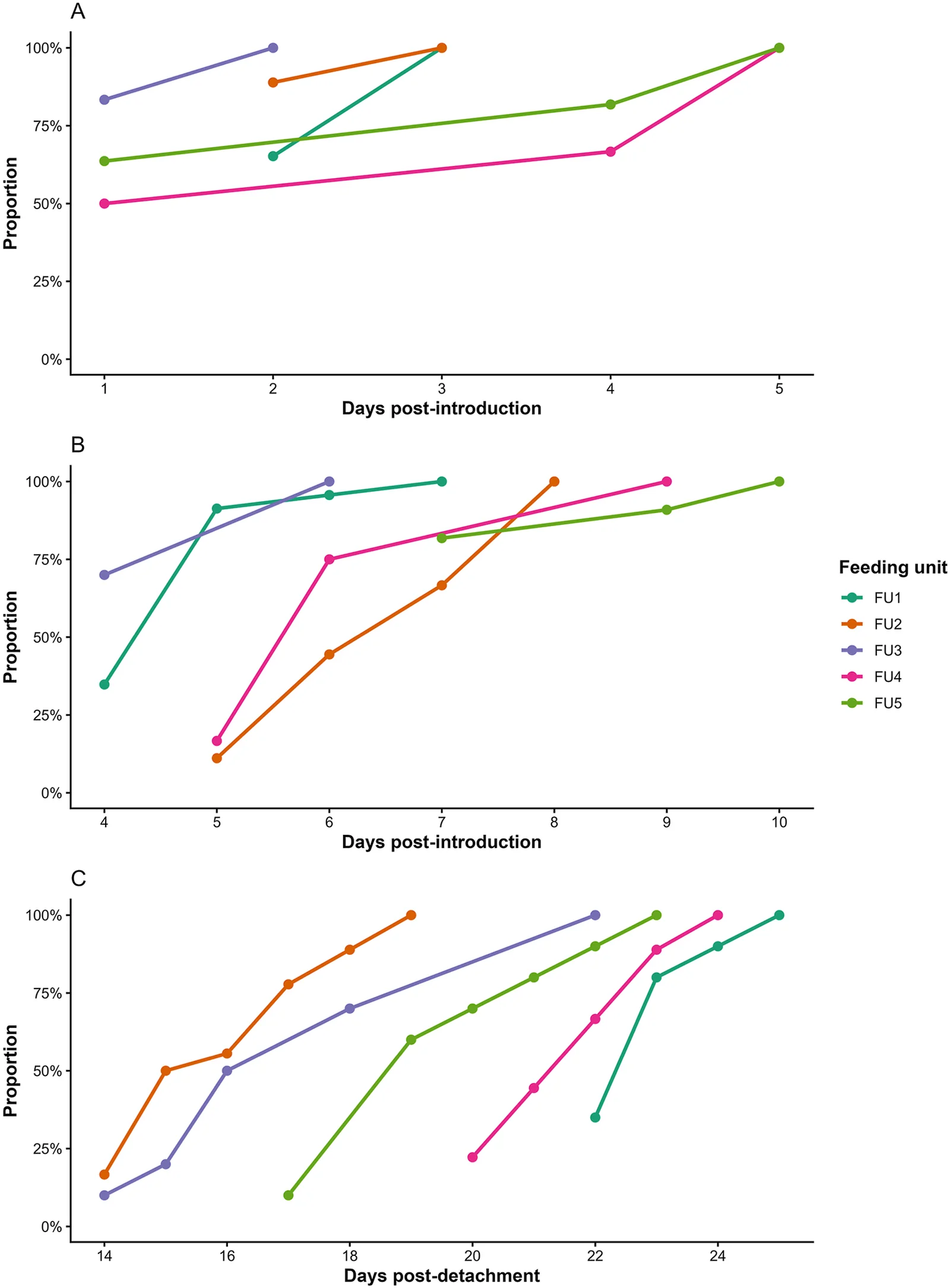
Temporal dynamics of feeding and development of *Haemaphysalis longicornis* nymphs across feeding units. **A** Cumulative attachment over time, showing rapid attachment within 1–3 days. **B** Cumulative detachment of engorged nymphs, occurring mainly between days 5–8 post-introduction. **C** Cumulative moulting post-detachment, showing greater variability than feeding duration. Lines represent individual feeding units.

Attachment occurred rapidly following introduction, with most nymphs attaching within 1–3 days across all feeding units and an overall attachment period of 1–5 days post-introduction (Table 1, Fig. 1, Fig. 2). Attachment timing differed significantly among feeding units (Kruskal-Wallis test, *χ*^2^ = 19.10, *df* = 4, *P* < 0.001), primarily because nymphs in FU3 attached earlier than those in FU1, FU2 and FU4 (Dunn’s test, Bonferroni-adjusted *P* < 0.05). Detachment of engorged nymphs occurred between 4 and 10 days post-introduction, with peak detachment observed between days 5 and 8. Detachment timing also differed significantly among feeding units (Kruskal-Wallis test, *χ*^2^ = 44.71, *df* = 4, *P* < 0.001), with several significant pairwise differences among feeding units (Dunn’s test, Bonferroni-adjusted *P* < 0.05). Moulting occurred between 14 and 25 days post-detachment, exhibiting greater variability than feeding duration both within and among feeding units. Moulting duration likewise differed significantly among feeding units (Kruskal-Wallis test, *χ*^2^ = 47.73, *df* = 4, *P* < 0.001), with earlier moulting in FU2, FU3 and FU5 than FU1, and in FU2 than FU4 (Dunn’s test, Bonferroni-adjusted *P* < 0.05). Despite significant differences in timing among feeding units, all units supported attachment, detachment of engorged nymphs and subsequent moulting within defined time windows.

Engorgement weights were recorded for individual nymphs where possible, with mean values ranging from 3.90 ± 1.10 mg in FU4 to 4.48 ± 0.65 mg in FU1 (Table 1). Engorgement weight did not differ significantly among feeding units (Kruskal-Wallis test, *χ*^2^ = 5.01, *df* = 4, *P* = 0.287), despite moderate within-unit variability. FU3 and FU4 tended to show lower mean weights, whereas FU1 and FU5 showed higher values, although variation within feeding units was substantial. Overall, engorgement weights were broadly comparable across feeding units, despite moderate within-group variability.

### 3.3 Microbiome composition across life stages

Amplicon sequencing generated a total of 4,070,337 paired-end reads across all samples and controls prior to processing. Following quality filtering, denoising, merging and chimera removal, 3,017,773 high-quality non-chimeric reads were retained overall. Biological samples accounted for 3,733,391 input reads, of which 2,870,698 non-chimeric reads were retained, corresponding to 76.9% retention after processing. Control samples accounted for 336,946 input reads, with 147,075 non-chimeric reads retained. Final non-chimeric read counts across biological samples ranged from 181,264 to 314,985 reads per sample, indicating consistently high sequencing depth.

To standardise sequencing results, samples were rarefied to 181,000 reads per sample, corresponding to the lowest read depth among biological samples; importantly, all biological samples were retained at this threshold (Supplementary Fig. S2). Observed richness ranged from 39 to 111 ASVs, with lower diversity in adults compared with unfed nymphs and larvae. Rarefaction analysis indicated sufficient sequencing depth for downstream alpha- and beta-diversity analyses.

**Fig. 3:**
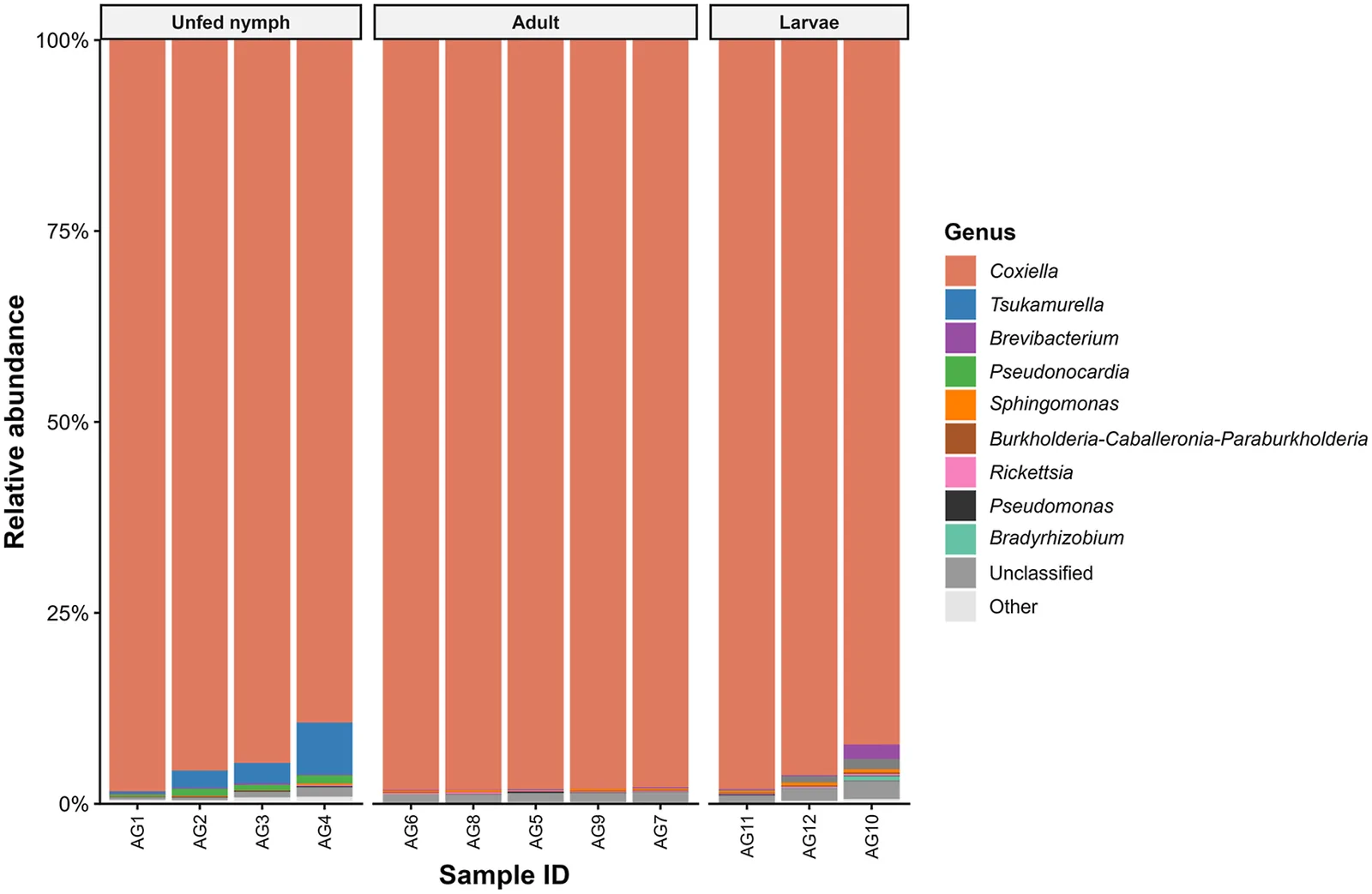
Relative abundance of the top ten bacterial genera across life stages (nymphs, adults and larvae). Remaining taxa are grouped as “Other.” Communities were strongly dominated by a *Coxiella*-like endosymbiont (> 95%), with only minor contributions from other genera.

**Fig. 4:**
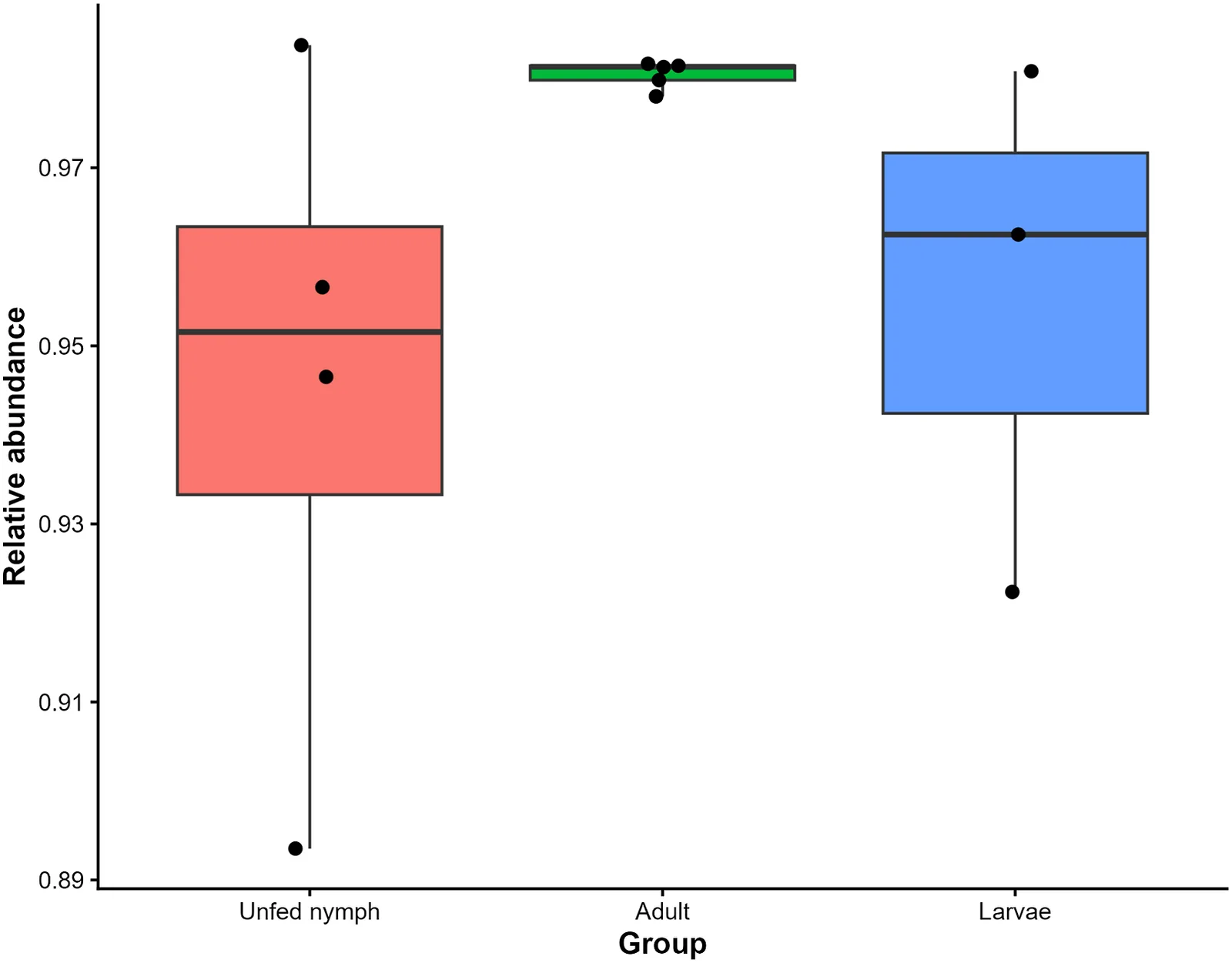
Relative abundance of *Coxiella* across life stages. Each point represents an individual sample. *Coxiella* dominated all stages, indicating strong persistence across development.

Microbiome profiling revealed a highly simplified bacterial community across all tick stages, dominated by a CLE (Fig. 3, Fig. 4). This taxon accounted for the majority of microbial sequences in unfed nymphs, post-fed adults and larvae, indicating a strong persistence across developmental stages. At the generic level, *Coxiella* overwhelmingly dominated in all life stages, accounting for 95.1% of reads in unfed nymphs, 99.2% in adults and 97.1% in larvae (Supplementary Table S1). In addition to *Coxiella*, low-abundance taxa were detected across samples, including *Tsukamurella*, *Brevibacterium*, *Pseudonocardia*, *Sphingomonas*, *Rickettsia* and *Pseudomonas*, although these collectively represented a small proportion (0.8–4.9%) of the overall community. Notably, all non-*Coxiella* genera occurred at < 3% relative abundance, and < 0.2% in adults, highlighting the highly simplified and symbiont-dominated nature of the microbiome. The overall *Coxiella*-dominated community structure was consistent across life stages, with no detectable shifts in the dominant CLE observed.

### 3.4 Alpha and beta diversity

**Fig. 5:**
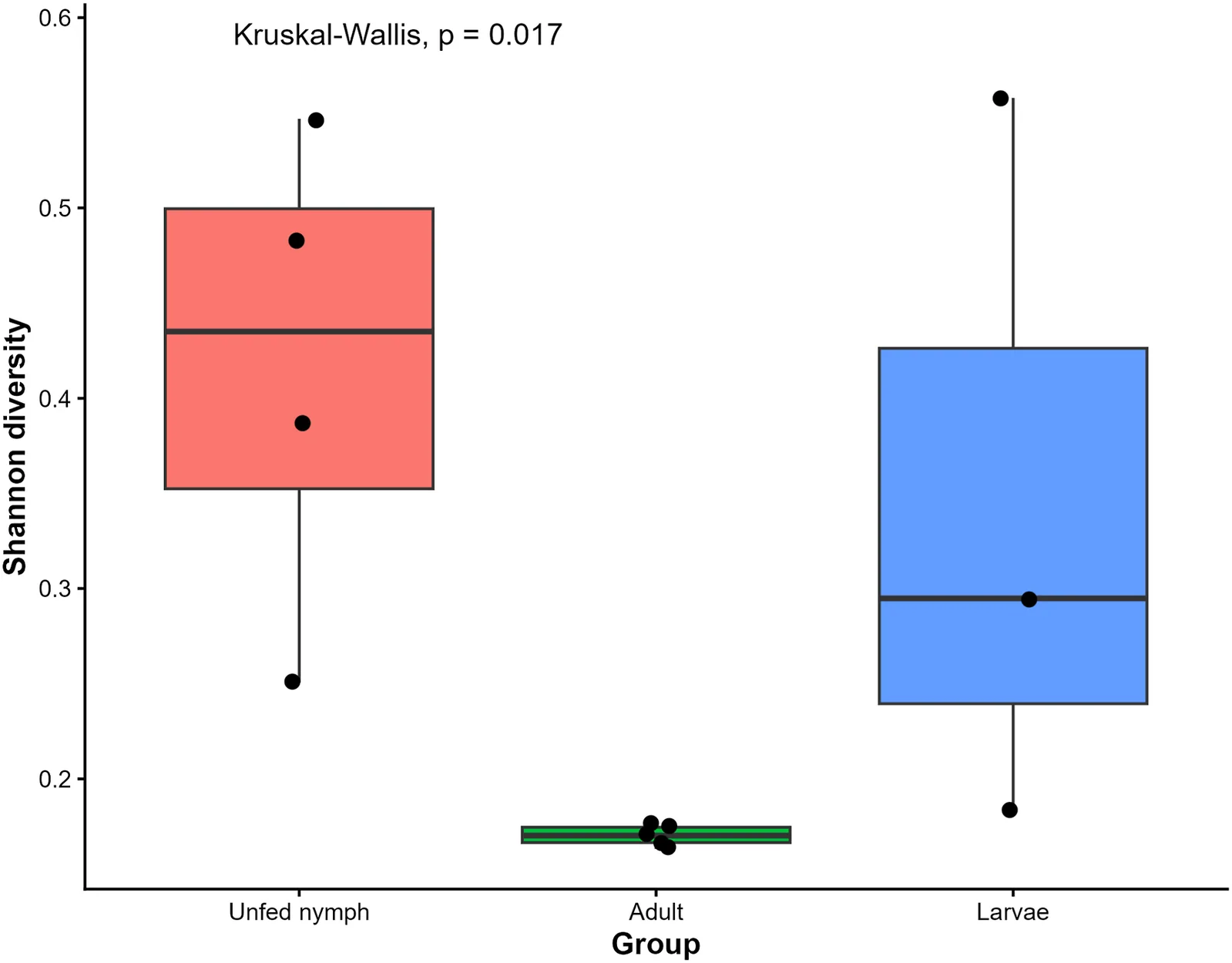
Alpha diversity of bacterial communities across life stages of *Haemaphysalis longicornis*, measured using the Shannon diversity index. Boxes represent interquartile ranges with median values, and whiskers indicate variability among samples.

Alpha diversity differed significantly among life stages for both Shannon diversity (Kruskal-Wallis test, *P* = 0.017) and observed richness (Kruskal-Wallis test, *P* = 0.0088) (Fig. 5, Supplementary Fig. S3). Pairwise comparisons indicated significantly lower diversity in adults compared to unfed nymphs for both Shannon diversity (Dunn’s test, adjusted *P* = 0.029) and observed richness (adjusted *P* = 0.0068), whereas no significant differences were detected between larvae and other life stages.

**Fig. 6:**
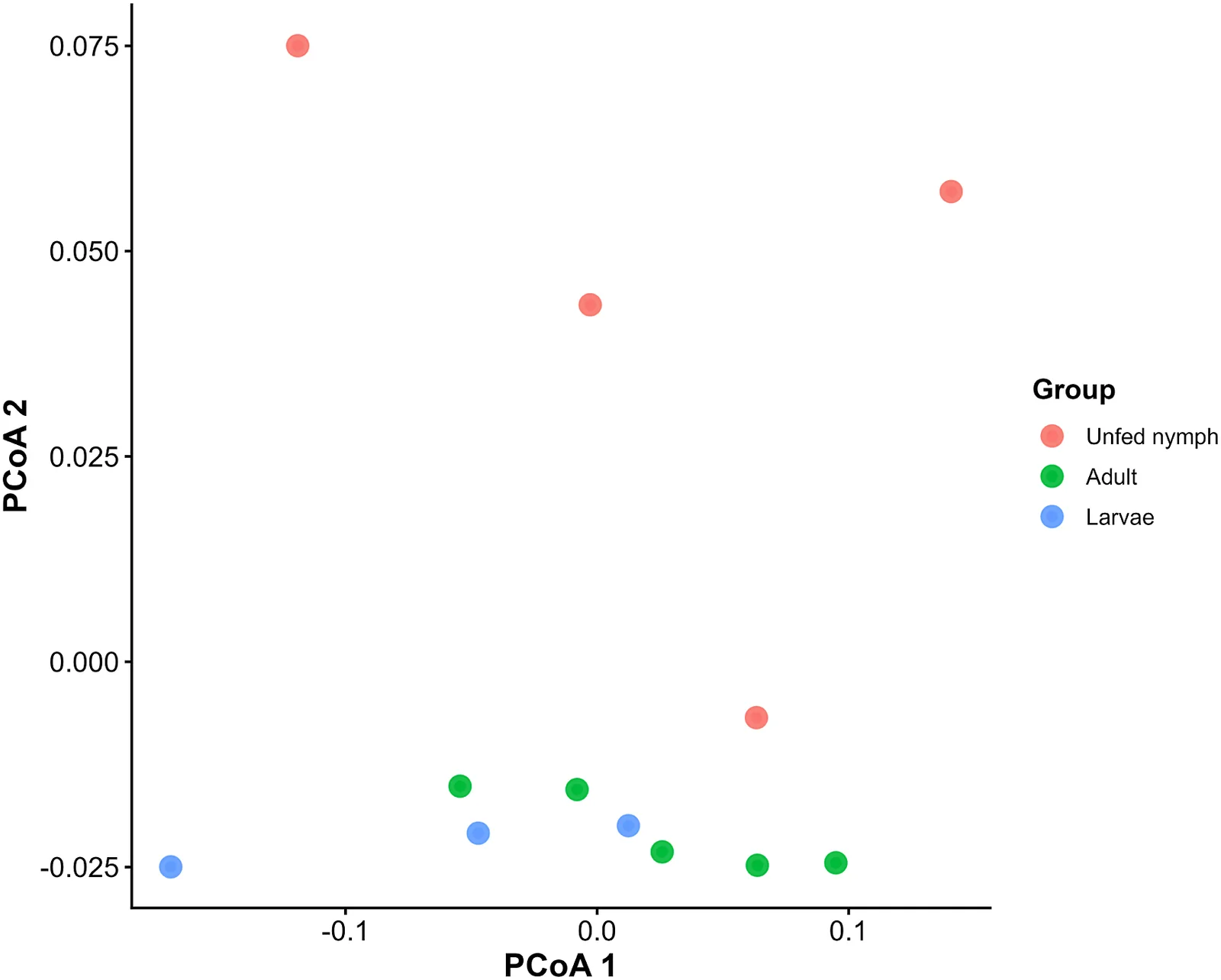
Beta diversity of bacterial communities across life stages of *Haemaphysalis longicornis*, visualised by principal coordinates analysis (PCoA) based on Bray-Curtis dissimilarity. Each point represents an individual sample, coloured by life stage.

Beta diversity analysis based on Bray-Curtis dissimilarity showed partial clustering of samples by life stage, although substantial overlap was observed among groups (Fig. 6). PERMANOVA did not detect significant differences in overall bacterial community composition among life stages (*P* > 0.05). Homogeneity of multivariate dispersion was also not significantly different among groups (PERMDISP, *P* = 0.496), indicating that within-group variability was comparable across life stages and that the lack of compositional differences was not influenced by dispersion effects. Given the small number of biological samples and the strong dominance of *Coxiella*, these results should be interpreted as evidence of no detectable large-scale community shift rather than definitive absence of microbiome variation among stages.

## 4 Discussion

Artificial tick feeding systems (ATFS) provide an important alternative to animal-based models, enabling controlled investigation of tick biology while supporting the principles of the 3Rs. In the present study, we successfully fed nymphal *H. longicornis* to repletion using a membrane-based system, achieving high attachment and engorgement rates, successful moulting to adults and maintenance of a *Coxiella*-dominated microbiome during prolonged maintenance without exposure to a live vertebrate host across life stages. These findings extend our previous work on adult *H. longicornis* (see Ghafar et al., 2026a) and represent an important advance toward establishing a complete *in vitro* life-cycle system for this invasive tick species.

High feeding performance was observed across all feeding units, with attachment rates exceeding 80% and complete engorgement achieved in most experiments. These values are comparable to, and in some cases exceed, those reported for other ixodid ticks under artificial feeding conditions (Kröber and Guerin, 2007; Nijhof and Tyson, 2018; Mustafa et al., 2026). Rapid attachment and predictable detachment timing further indicate that key behavioural and physiological aspects of feeding were effectively reproduced under *in vitro* conditions. The high feeding success likely reflects optimisation of environmental parameters within the ATFS, including temperature, humidity and CO_2_ levels that mimic natural host-associated conditions.

Despite consistently high feeding success, variability was observed in post-feeding development, particularly moulting duration. Similar variability has been reported previously and is likely influenced by differences in bloodmeal acquisition, nutritional status and intrinsic physiological regulation during the nymph-to-adult transition (Belozerov, 1982; Ogden et al., 2004; Sonenshine and Roe, 2014; Militzer et al., 2021; Elati et al., 2024; Erguler et al., 2025). Because ticks in this study were maintained under controlled conditions, the observed variation likely reflects genuine individual-level biological heterogeneity rather than environmental noise. Nevertheless, the high overall moulting success across feeding units indicates that the ATFS provides sufficient nutritional and physiological support for completion of development to the adult stage. As all ticks originated from a single field population maintained under uniform laboratory conditions, caution is warranted when extrapolating these findings to broader populations.

Engorgement weights were broadly comparable across feeding units (3.90–4.48 mg), with moderate variation observed within and among groups (SD: 0.48–1.10 mg). Although no statistically significant differences were detected, the observed range in weights indicates heterogeneity in individual feeding efficiency. The mean engorgement weight observed in the present study (4.23 ± 0.77 mg) was somewhat higher than previously reported field-fed *H. longicornis* nymphs (3.03 ± 0.90 mg) but remained within a biologically plausible range (Zheng et al., 2011). Such variability has been attributed to differences in attachment timing, feeding duration and host-parasite interaction dynamics (Kröber and Guerin, 2007; Mustafa et al., 2026). This heterogeneity may also reflect intrinsic behavioural and physiological variation, as *H. longicornis* exhibits pronounced diel activity patterns and temperature-dependent peaks in host-seeking behaviour under natural conditions (Noh et al., 2024).

A central aim of this study was to determine whether artificial feeding and prolonged maintenance without live-host exposure influence the tick microbiome. Across unfed nymphs, adults moulted following *in vitro* feeding, and larval progeny, the microbiome was overwhelmingly dominated by a CLE, with only minor contributions from other taxa. This pattern is consistent with previous reports identifying CLEs as dominant endosymbionts of *H. longicornis* and other ixodid ticks (Noda et al., 1997; Liu et al., 2013; Duron et al., 2015; Bonnet et al., 2017). These symbionts are vertically transmitted and are thought to contribute essential nutritional functions, including B vitamin biosynthesis required for tick development and reproduction (Smith et al., 2015; Kusakisako et al., 2026). Experimental disruption of *Coxiella* symbionts has also been associated with impaired feeding, reproduction and offspring viability (Zhang et al., 2017), supporting their importance in tick biology.

Alpha diversity was significantly lower in adults than in unfed nymphs, indicating a reduction in microbial diversity following feeding and development. This pattern is consistent with studies showing that blood-feeding acts as a selective bottleneck, favouring the proliferation of essential symbionts while reducing overall diversity (Narasimhan and Fikrig, 2015; Bonnet et al., 2017). The reduction in diversity observed here may reflect selective enrichment and maintenance of dominant symbionts, particularly *Coxiella*, following feeding and development. The absence of significant differences between larvae and other stages may reflect re-establishment of microbial communities following oviposition, although this interpretation should be considered cautiously given the relatively small sample size.

In contrast to the preservation observed in the present study, field-based investigations of *H. longicornis* have reported pronounced restructuring of microbial communities across developmental stages, with significant differences in both alpha and beta diversity between larvae, nymphs and adults (Ponnusamy et al., 2024). These shifts have been attributed to environmental exposure, variation in host bloodmeal sources, and ecological interactions that influence microbial acquisition and turnover. The absence of major microbiome restructuring under prolonged *in vitro* conditions suggests that ATFS may buffer external ecological influences, enabling persistence of a dominant *Coxiella*-associated microbiome despite extended starvation.

Notably, DNA extractions for this study were performed several months after feeding, providing evidence that the dominant CLE and overall low-diversity community structure can persist during extended *in vitro* maintenance. Given that ticks can undergo prolonged periods of metabolic suppression during starvation, the observed microbiome composition may also reflect tightly regulated host-microbe interactions maintained under low metabolic activity, rather than continuous microbial turnover. This suggests that the tick microbiome is not passively maintained but actively preserved as part of a stable physiological system during extended periods without exposure to a live host. Together, these findings indicate that the ATFS not only reduces environmental variability but preserves a biologically regulated core symbiotic system driven by strong host-symbiont dependence (Bonnet et al., 2017). Collectively, these findings suggest that ATFS-derived ticks retain core microbiome characteristics across life stages despite prolonged maintenance in the absence of a vertebrate host.

This study has some limitations. Microbiome analyses were based on a modest number of samples, and sample types differed among life stages, with pooled nymph and larval samples compared with individual adults. Notably, this study did not include a parallel group fed on a live vertebrate host, so the extent to which artificial feeding reproduces *in vivo* feeding parameters and microbiome structure remains to be confirmed; incorporating a naturally fed control will be an important step in future work. In addition, all ticks originated from a single field population, which strengthens internal consistency but limits inference across genetically diverse or geographically distant populations. The dominance of *Coxiella* also reduces sensitivity for detecting subtle changes in low-abundance taxa. Finally, 16S rRNA sequencing provides taxonomic rather than functional resolution. Therefore, future metagenomic, transcriptomic and symbiont-manipulation studies will be required to investigate functional host-symbiont interactions under artificial feeding conditions.

Collectively, the findings of this study demonstrate that ATFS can support nymphal feeding, development to adults and maintenance of a *Coxiella*-dominated microbiome under prolonged *in vitro* conditions. Establishment of reliable feeding and moulting of immature stages represents an important advance toward a complete *in vitro* life-cycle system for *H. longicornis* and provides a controlled platform for investigating tick physiology, symbiont biology, pathogen interactions and intervention strategies under standardised experimental conditions.

## 5 Conclusions

This investigation demonstrates that nymphal *H. longicornis* can be reliably fed to repletion using a fully artificial membrane feeding system that does not require a live vertebrate host, with successful moulting to adults. The persistence of a strongly *Coxiella*-dominated microbiome across unfed nymphs, *in vitro*-fed adults and larval progeny indicates that core host-symbiont associations are retained under prolonged maintenance in the absence of a live host. Together, these findings represent a key step toward establishing a complete *in vitro* life-cycle system for *H. longicornis* and provide a biologically relevant platform for future studies of tick physiology, symbiont function, pathogen interactions and control strategies while advancing the principles of the 3Rs in tick research.

## Ethical approval

Not applicable. This study was conducted entirely using an *in vitro* artificial membrane feeding system and did not involve the experimental use of live vertebrate animals. Consequently, animal ethics approval was not required.

## CRediT authorship contribution statement

**Abdul Ghafar**: Writing - original draft, Visualisation, Validation, Methodology, Investigation, Funding acquisition, Formal analysis, Data curation, Conceptualisation. **Bahar E. Mustafa**: Investigation, Writing - review & editing. **Mehran Khan**: Writing - review & editing. **Charles Gauci**: Writing - review & editing. **Ian Beveridge**: Writing - review & editing. **Robin B. Gasser**: Writing - review & editing. **Ard M. Nijhof**: Writing - review & editing, Methodology, Funding acquisition, Conceptualisation. **Abdul Jabbar**: Writing - review & editing, Resources, Project administration, Methodology, Funding acquisition, Conceptualisation.

## Funding

This work was supported by the Melbourne Postdoctoral Fellowship awarded to Abdul Ghafar by the University of Melbourne under the project “Toward Next-Generation Interventions Against Ticks and Tick-Borne Diseases at a Critical Time of Climate Change” (Grant No. 503706). Abdul Jabbar and Ard M. Nijhof acknowledge funding support from the Berlin University Alliance and the University of Melbourne, which facilitated collaborative work related to this study.

## Declaration of competing interests

The authors declare that they have no competing interests. Given their role as Co-Editor, Abdul Jabbar had no involvement in the peer review of this article and has no access to information regarding its peer review. Full responsibility for the editorial process for this article was delegated to Professor Aneta Kostadinova (Editor-in-Chief).

## Data Availability

All relevant data generated or analysed during this study are included in this published article and its supplementary information files.
