# Supplementary material for: In vitro feeding of nymphal Haemaphysalis longicornis supports moulting to adults and maintains Coxiella-dominated microbiome across life stages
Source: Curr Res Parasitol Vector Borne Dis. 2026 Aug 18;10:100426. doi: 10.1016/j.crpvbd.2026.100426 (PMC13520983; doi:10.1016/j.crpvbd.2026.100426)
Supplement: Supplementary file 1 [file mmc1.pdf]

Supplementary figures

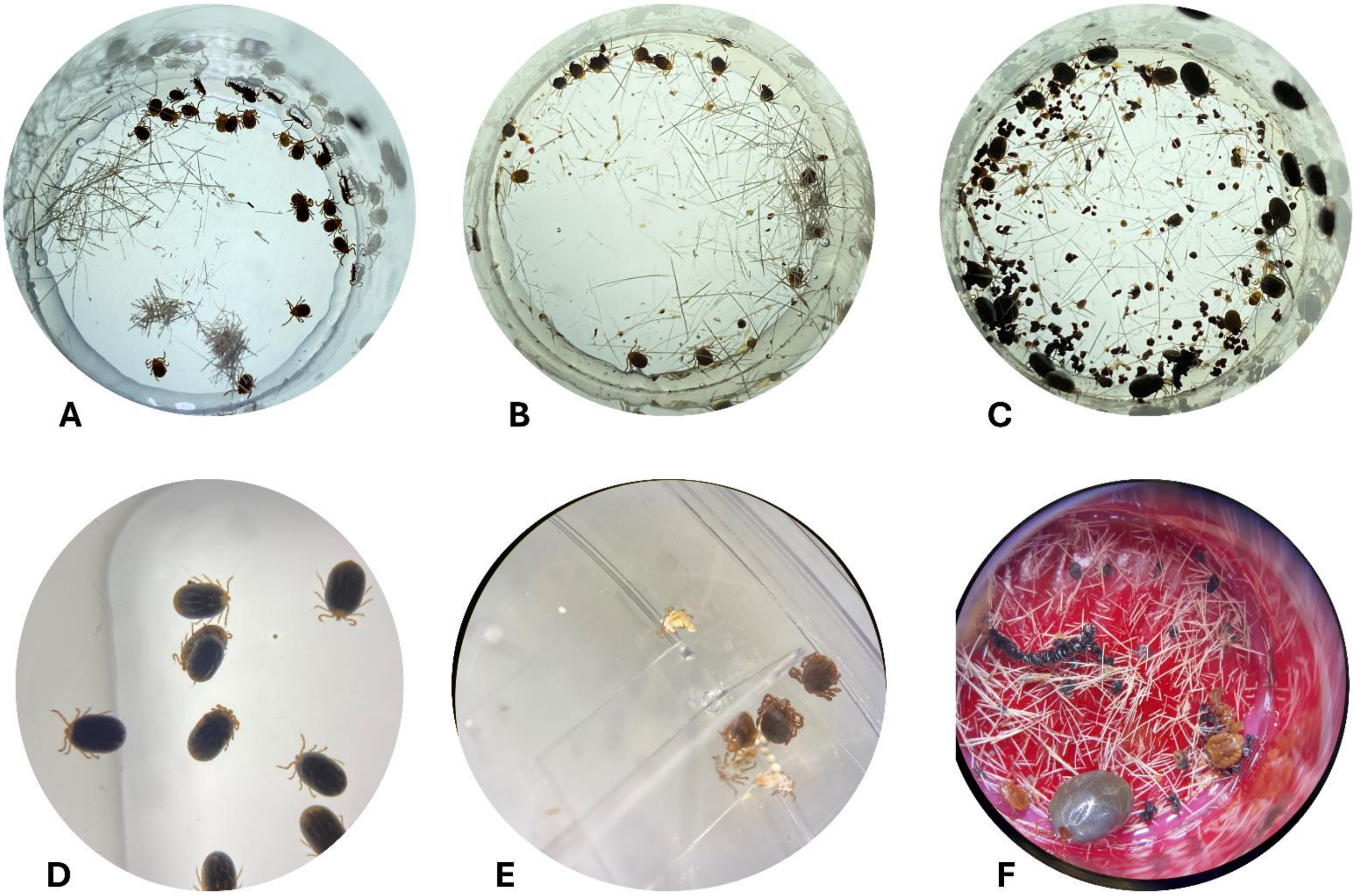

Supplementary Figure S1. Representative stages of *in vitro* feeding and development of *Haemaphysalis longicornis*. Panels show (**A**) pre-attachment nymph, (**B**) early attachment to the membrane, (**C**) engorging nymph, (**D**) detached engorged nymph, (**E**) moulted adult, and (**F**) engorged adult following *in vitro* feeding.

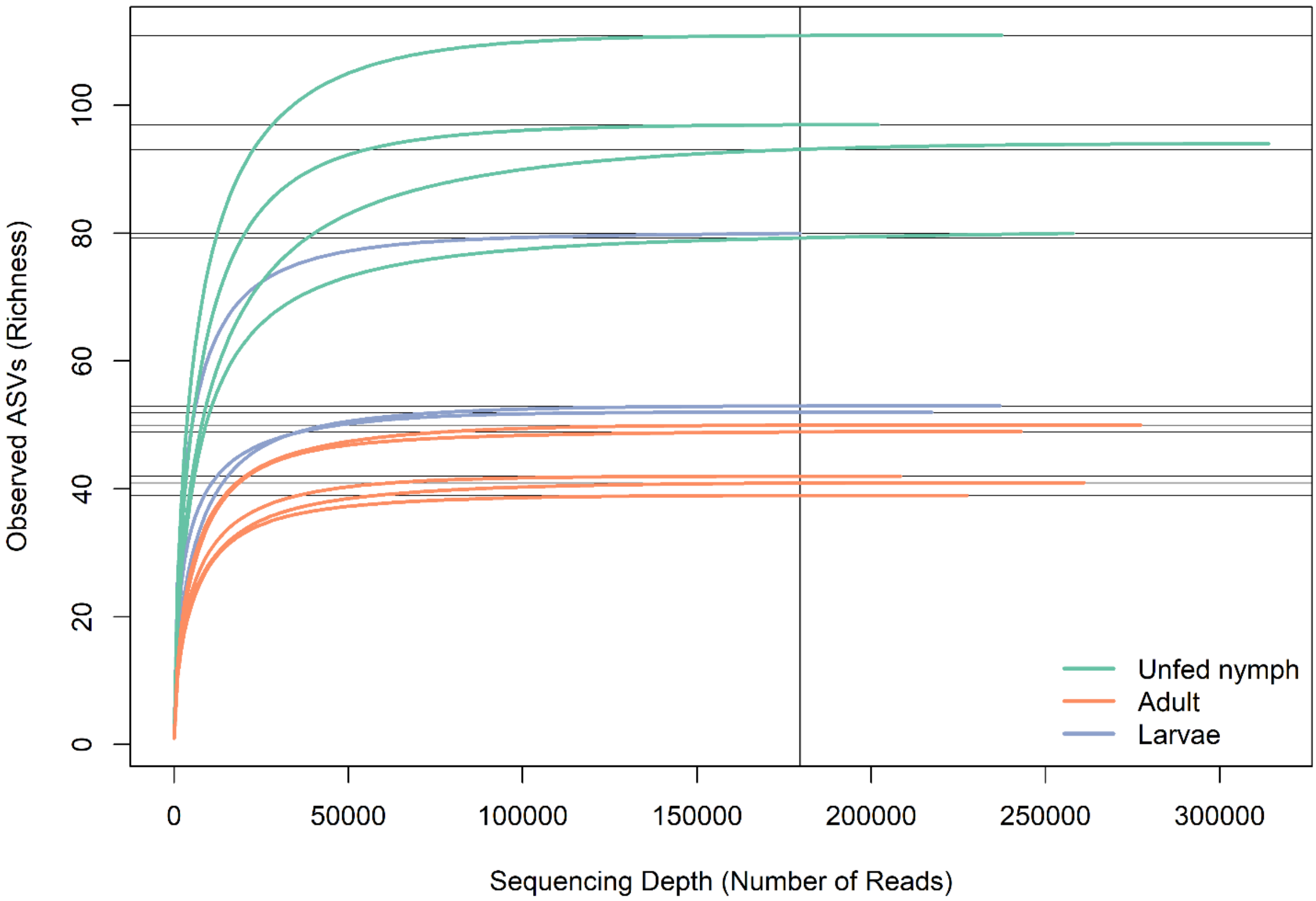

Supplementary Figure S2. Rarefaction curves showing observed ASV richness as a function of sequencing depth across all samples. Each curve represents an individual sample, coloured by life stage. Curves approached saturation, indicating sufficient sequencing depth for downstream diversity analyses. The vertical line indicates the rarefaction threshold (181,000 reads per sample) used for standardisation.

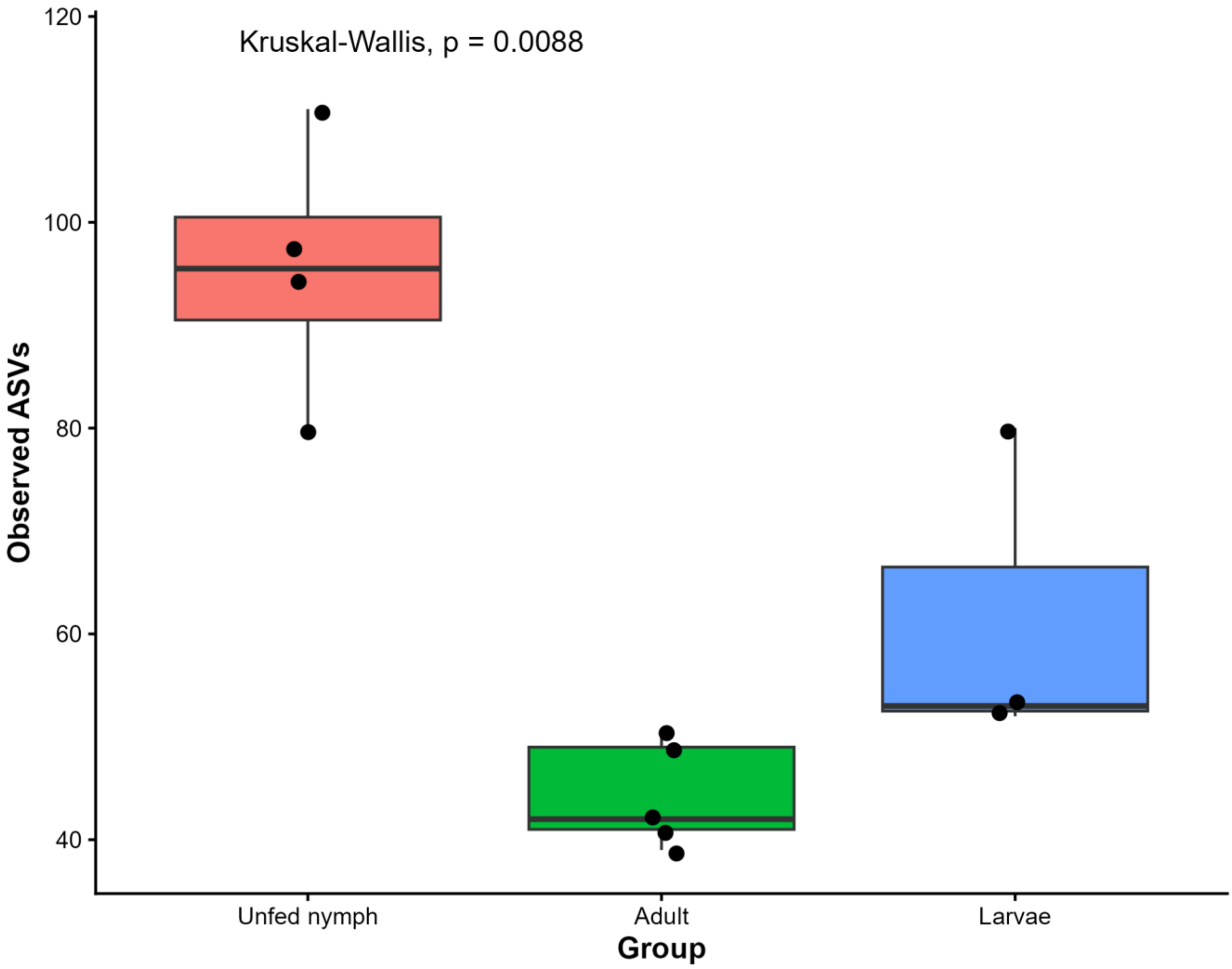

Supplementary Figure S3. Alpha diversity of bacterial communities across life stages of *Haemaphysalis longicornis*, measured using observed ASV richness. Boxes represent interquartile ranges with median values, and whiskers indicate variability among samples. Significant differences among life stages were detected using the Kruskal-Wallis test (P = 0.0088).
