# Supplementary material for: In vitro feeding of nymphal Haemaphysalis longicornis supports moulting to adults and maintains Coxiella-dominated microbiome across life stages
Source: Curr Res Parasitol Vector Borne Dis. 2026 Aug 18;10:100426. doi: 10.1016/j.crpvbd.2026.100426 (PMC13520983; doi:10.1016/j.crpvbd.2026.100426)
Supplement: Supplementary file 2 [file mmc2.pdf]

Supplementary Table S1

Mean relative abundance (%) of dominant bacterial genera across unfed nymphs, adults and larvae of *Haemaphysalis longicornis* based on rarefied 16S rRNA gene sequencing data. Relative abundances were calculated from rarefied data (181,000 reads per sample) and averaged within each life stage.

**Genus**	**Unfed nymph (%)**	**Adult (%)**	**Larvae (%)**
Coxiella	95.10	99.20	97.10
Tsukamurella	3.010	0.0005	0.0019
Brevibacterium	0.140	0.100	0.71
Sphingomonas	0.094	0.176	0.36
Rickettsia	0.030	0.122	0.148
Pseudonocardia	0.730	0.003	0.001
Pseudomonas	0.065	0.060	0.069
Stenotrophomonas	0.032	0.045	0.091
Burkholderia complex	0.058	0.090	0.166
Methylobacterium	0.043	0.008	0.060
Acinetobacter	0.030	0.009	0.026
Bradyrhizobium	0.031	0.001	0.215
Dietzia	0.046	0.007	0.020
Nocardiopsis	0.033	0.007	0.017
Escherichia-Shigella	0.038	0.007	0.013
